# Mortality after major lower extremity amputation and association with index level: a cohort study based on 11,205 first-time amputations from nationwide Danish databases

**DOI:** 10.2340/17453674.2024.40996

**Published:** 2024-06-19

**Authors:** Anna Trier Heiberg BRIX, Katrine Hass RUBIN, Tine NYMARK, Hagen SCHMAL, Martin LINDBERG-LARSEN

**Affiliations:** 1 Department of Orthopedic Surgery and Traumatology, Odense University Hospital, Odense; 2 Department of Clinical Research, University of Southern Denmark, Odense; 3 OPEN – Open Patient Data Explorative Network, Odense University Hospital and University of Southern Denmark, Odense; Denmark; 4 Department of Orthopedics and Traumatology, University Medical Center Freiburg, Germany

## Abstract

**Background and purpose:**

Mortality after major lower extremity amputations is high and may depend on amputation level. We aimed to examine the mortality risk in the first year after major lower extremity amputation divided into transtibial and transfemoral amputations

**Methods:**

This observational cohort study used data from the Danish Nationwide Health registers. 11,205 first-time major lower extremity amputations were included from January 1, 2010, to December 31, 2021, comprising 3,921 transtibial amputations and 7,284 transfemoral amputations.

**Results:**

The 30-day mortality after transtibial amputation was overall 11%, 95% confidence interval (CI) 10–12 (440/3,921) during the study period, but declined from 10%, CI 7–13 (37/381) in 2010 to 7%, CI 4–11 (15/220) in 2021. The 1-year mortality was 29% overall, CI 28–30 (1,140 /3,921), with a decline from 31%, CI 21–36 (117/381) to 20%, CI 15–26 (45/220) during the study period. For initial transfemoral amputation, the 30-day mortality was overall 23%, CI 22–23 (1,673/7,284) and declined from 27%, CI 23–31 (138/509) to 22%, CI 19–25 (148/683) during the study period. The 1-year mortality was 48% overall, CI 46–49 (3,466/7,284) and declined from 55%, CI 50–59 (279/509) to 46%, CI 42–50 (315/638).

**Conclusion:**

The mortality after major lower extremity amputation declined in the 12-year study period; however, the 1-year mortality remained high after both transtibial and transfemoral amputations (20% and 46% in 2021). Hence, major lower extremity amputation patients constitute one of the most fragile orthopedic patient groups, emphasizing an increased need for attention in the pre-, peri-, and postoperative setting.

Major lower extremity amputations are performed on frail patients with an extensive comorbidity profile [1-2]. These amputations, often necessitated by vascular compromise or complex systemic diseases, have frequently yielded suboptimal outcomes despite the general advancements in perioperative care [3-5]. This is mirrored in the high postoperative mortality, depending on the index level [3,6]. The 30-day mortality rates after a below-knee amputation may vary from 5% to 12% and range from 13% to 22% after above-knee amputation [4,7,8]. Furthermore, the 1-year mortality risks were reported at 23–33% for below-knee amputation and 41–58% for above-knee amputation [3,8].

The index level can be divided into transtibial amputation, knee disarticulation, transfemoral amputation, and hip disarticulation. Transtibial and transfemoral amputation consists of 95% of the initial major lower extremity amputations in Denmark and a trend towards a more proximal initial amputation level has been observed (Personal communication, Brix et al. 2023).

Many factors contribute when the initial amputation level is decided; the mortality after an above-knee amputation is known to be higher, but the risk of re-amputation is lower at a more proximal level [9].

We aimed to examine the mortality risk in the first year after major lower extremity amputation divided into transtibial and transfemoral amputations during a 12-year period from 2010–2021. Furthermore, we examined time-trends in mortality risk.

## Method

This was an observational nationwide cohort study. REporting of studies Conducted using Observational Routinely-collected health Data (RECORD) guidelines were followed [10].

### Data sources

The study was based on data from the National Danish Health registers. The Danish National Patient Registry (DNPR) provided ICD-10 diagnoses and NOMESCO surgical procedures from 1977 to present day from all Danish hospitalizations [11]. Patients’ unique 10-digit social security number served as identifier, enabling successful linkage between registers [12]. Data from the DNPR has an estimated data completeness of > 99%; however, differences in the validity of the diagnosis codes in the registry have been reported [11].

The data from the Danish National Prescription Database (DNPD) held all reimbursed prescriptions by Danish citizens starting from 1995. The medications prescribed were classified according to Anatomical Therapeutic Chemical (ATC) codes [13].

The Danish Civil Registration System contained data on date of birth, date of death, region of living, and marital status [12].

### Study population

The population included first-time major amputation procedures performed in Denmark from January 1, 2010, to December 31, 2021 acquired from the DNPR. The index procedure was the first major amputation, either transtibial or transfemoral, performed on the right or left leg in patients aged 50 years or older. Cases involving initial knee disarticulation, initial hip disarticulation, solely revision amputation procedures, cases diagnosed with sarcoma or with a registered trauma in relation to amputation were excluded (see Supplementary data). In instances where multiple amputation procedures were documented on the same day on the same extremity, the most proximal level was registered as index level.

To ensure that only first-time major lower extremity amputation was included, the Danish Health Data Authority excluded all patients with a prior procedure code for amputation on thigh/hip (KNFQ*) and amputation on lower leg/knee (KNGQ*) in 1996–2009, from the dataset. Amputation procedures in the DNPR are not validated.

### Outcomes

The primary outcome was 1-year mortality risk, but also 30- and 90-day mortality risks were reported.

### Covariates

Sex, age at surgery, and registered diagnoses were obtained from the DNPR. Data from the DNPR was used to calculate the Charlson comorbidity index (CCI) with data 10 years prior to inclusion, with the method from Quan et al. [14]. The CCI score was classified into 3 groups: low (CCI score of 0), medium (CCI score 1–2) or high (CCI score ≥ 3).

DNPR data was used to support the definition of selected comorbidities: dyslipidemia, diabetes, and hypertension. The DNPR contains only diagnoses acquired in a hospital, while patients treated by their general practitioner are not listed in the DNPR [13]. Therefore, e.g., dyslipidemia was considered present if the ICD10-code E78 was registered or the patient had redeemed 2 or more prescriptions in the same ATC group (C10, statins) 5 years before the index surgery.

For all defined comorbidities, see Supplementary data.

### Statistics

Results were reported as medians with interquartile range (IQR) for non-normally distributed data. Categorical data was presented with actual number (%). Risks were calculated as cases with event (mortality) divided by the total number of cases at a given time, both overall and as time trends.

Cox regression analysis was used to address the difference in mortality depending on initial amputation level. Hazard ratios (HRs) were adjusted for all included comorbidities (diabetes, peripheral arterial disease, cardiovascular disease, hypertension, dyslipidemia, renal insufficiency, prior minor amputation, prior revascularization and CCI score), sex, and age, and reported as HRs with 95% confidence intervals (CI). The HR between transfemoral amputation and transtibial amputation was reported for 30-day, 90-day, and 1-year mortality. Initial transtibial amputation was the reference in all analyses. Model assumptions were checked with the proportional hazard test using Schoenfeld residuals and were non-significant at the 30-day period but significant for the 90-day and 1-year period. The Schoenfeld residuals were plotted against time and examined for any systematic patterns, indicating violation of the proportional hazard assumption. The visual inspection suggested that the proportional hazards assumption was met. All analyses were conducted in STATA v17.0 (2021; StataCorp LLC, College Station, TX, USA).

### Ethics, data sharing, funding, and disclosures

The study was approved by the Danish Data Protection Agency in the Region of Southern Denmark (no. 21/27110). Because of the observational study design, ethical approval was not relevant. Funding for this study was obtained from the Region of Southern Denmark and Odense University Hospital. Data is accessible through the Danish Health Data Authority, but is not publicly available, therefore raw data for this study cannot be shared.

No artificial intelligence tools were systematically used to generate whole sections within this manuscript; however, ChatGPT was used for minor text editing. The authors have no competing interests to declare. Complete disclosure of interest forms according to ICMJE are available on the article page, doi: 10.2340/17453674.2024.40996

## Results

**Figure 1:**
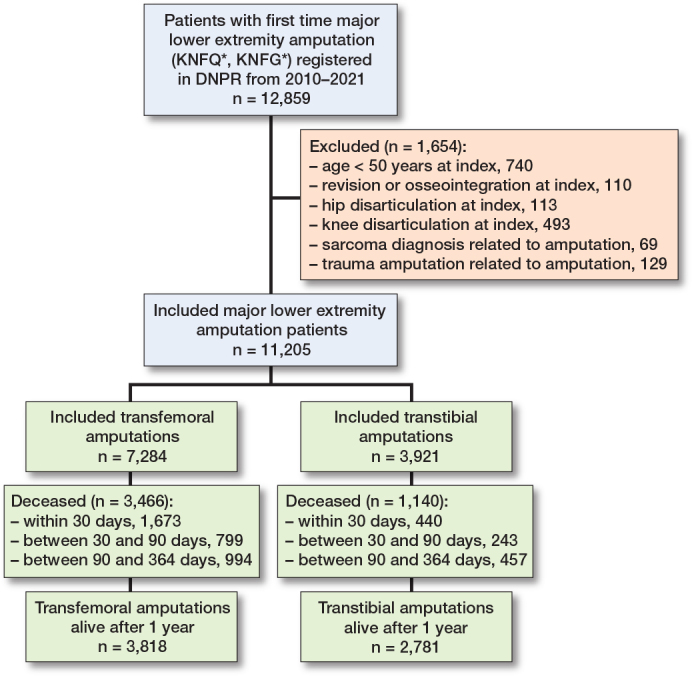
Flowchart of included patients. DNPR: Danish National Patient Register.

12,859 major lower extremity amputations were identified and 11,205 cases were eligible for inclusion, divided into 7,284 transfemoral amputations and 3,921 transtibial amputations (Figure 1). Median age at transtibial amputation was 71.7 years (IQR 64–79), while it was 77.2 years (IQR 70–84) at transfemoral amputation. 70% of the transtibial amputees and 55% of the transfemoral amputees were men (Table 1). The distribution of comorbidities showed a higher frequency of diabetes, renal insufficiency, prior minor amputation (toe, foot, and Symes amputation) and revascularization procedures in the transtibial group. The distribution of CCI scores was uneven between groups with fewer patients with CCI score 0, and more in the CCI score 1–2, in the transtibial group than the transfemoral group.

**Table 1:** Baseline characteristics for the 11,205 included major lower extremity amputations patients. Values are count (%) unless otherwise specified

	Index amputation level	
	transtibial	transfemoral
Total number	3,921 (35)	7,284 (65)
Age, median (IQR)	71.7 (64–79)	77.2 (70–84)
Age group		
50–70	1,716 (44)	1,860 (26)
71–80	1,311 (33)	2,505 (34)
81–90	747 (19)	2,259 (31)
> 90	147 (3.7)	660 (9.1)
Male sex	2,754 (70)	3,995 (55)
Married	2,380 (61)	4,137 (57)
Comorbidity		
Diabetes	2,392 (61)	2,660 (36)
Peripheral arterial disease	3,193 (81)	6,054 (83)
Cardiovascular disease	1,629 (41)	2,817 (39)
Hypertension	3,462 (88)	6,437 (88)
Dyslipidemia	2,714 (69)	4,468 (61)
Renal insufficiency	760 (19)	1,030 (14)
Prior surgery		
Minor amputation	1,429 (36)	855 (12)
Revascularization	1,766 (45)	2,786 (38)
Charlson comorbidity index		
0	766 (19)	2,221 (31)
1–2	1,905 (49)	2,874 (39)
≥ 3	1,250 (32)	2,189 (30)

### Postoperative mortality risk

The mortality risk after major amputation declined for both transtibial amputation and transfemoral amputation during the study period at all investigated time points: 30 days, 90 days, and 1 year.

***Transtibial amputations.*** The overall mortality after transtibial amputation was 11% (CI 10–12), 17% (CI 16–19), and 29% (CI 28–30) within 30 days, 90 days, and 1 year respectively (Table 2).

**Table 2:** Overall 30-day, 90-day, and 1-year mortality after major lower extremity amputation, divided into index levels

	Deaths, n (% [CI])		Cox regression	
	Transtibial amputation (n = 3,921)	Transfemoral amputation (n = 7,284)	Reference: transtibial amaputation	
			Adjusted^a^ HR (CI)	Crude HR (CI)
30 days	440 (11 [10–12])	1,673 (23 [22–24])	1.8 (1.7–2.1)	2.2 (1.9–2.4)
90 days	683 (17 [16–18])	2,472 (34 [33–35])	1.8 (1.6–1.9)	2.2 (2.0–2.4)
1 year	1,140 (29 [28–30])	3,466 (47 [46–49])	1.6 (1.5–1.7)	1.9 (1.8–2.1)

HR: hazard ratio, Cox regression on mortality risk depending on index level with transtibial amputation as reference.

a The hazard ratios are adjusted for comorbidities (i.e., diabetes, peripheral arterial disease, cardiovascular disease, hypertension, dyslipidemia, renal insufficiency, prior minor amputation, prior revascularization and CCI score), age, and sex.

**Figure 2:**
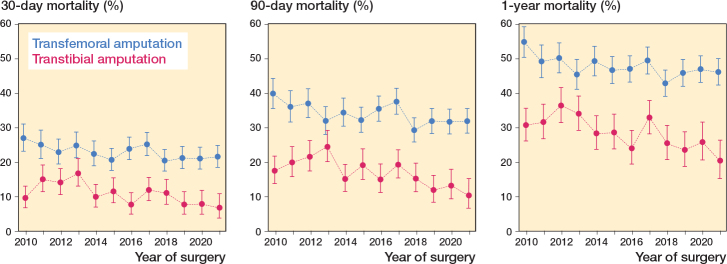
Changes in 30-day, 90-day, and 1-year mortality after major lower extremity amputations from 2010–2021.

The 30-day mortality declined from 10% (CI 7–13) in 2010 to 7% (CI 4–11) in 2021 (Figure 2 and Table 3, see Supplementary data). The 90-day mortality declined from 18% (CI 14–22) in 2010 to 10% (CI 7–15) in 2021, while 1-year mortality declined from 31% (CI 21–36) to 20% (CI 15–26) within the 12-year study period.

***Transfemoral amputations.*** The overall mortality after transfemoral amputation was 23% (CI 22–23), 34% (CI 33–35), and 48% (CI 46–49) within 30 days, 90 days, and 1 year respectively (Table 2).

The 30-day mortality declined from 27% (CI 23–31) in 2010 to 22% (CI 19–25) in 2021 (Figure 2 and and Table 3, see Supplementary data). The 90-day mortality declined from 40% (CI 35–44) to 32% (CI 28–35), and the 1-year mortality declined from 55% (CI 50–59) to 46% (CI 42–50).

When adjusting for age, sex, and comorbidities the risk of mortality was significantly higher after transfemoral amputation compared with transtibial amputation (Table 2).

## Discussion

Our study investigated the mortality risk following major lower extremity amputations over time for both initial transtibial and transfemoral amputations. We found that the mortality risk decreased over the study period for both amputation levels, but remained significantly higher after transfemoral amputations.

### Postoperative mortality risk

The findings of this study are consistent with previously reported mortality risks following major lower extremity amputation [3,6-8].

Our investigation into the mortality risk from 2010 to 2021 revealed an overall declining trend in mortality after major amputation at 30 days, 90 days, and 1 year. The overall decline in mortality may be because of development in pre-, peri-, and postoperative care. The decrease in mortality risk following transtibial amputations could be explained by a reduction in transtibial amputation procedures or a change in the selection process for patients undergoing initial transtibial amputation, as a trend towards a more proximal index amputation level has been observed (Personal communication, Brix et al. 2023).

As evidenced by studies [5,15], local initiatives have been implemented to improve outcomes following major amputation in Denmark. It was conceivable to reduce mortality within a specific ward by placing extended emphasis on this patient group, particularly through early mobilization [4,5]. However, it did not lead to the same mortality reduction nationwide.

Despite the observed reduction in mortality risk from 2010 to 2021, mortality following major amputation remained high, potentially ranking among the highest within the orthopedic patient cohort. For comparison, mortality risks after hip fractures have been reported to be 9.^7^%, 16%, and 27%, at 30 days, 90 days, and 1 year respectively [16]. These figures were almost equivalent to the mortality risk after a transtibial amputation, despite the patients being younger than hip fracture patients. In contrast, transfemoral amputees exhibited significantly higher mortality risks, with rates surpassing those of some malignant diseases [17].

Patients undergoing major lower extremity amputation present with an extensive comorbidity profile, often necessitating the amputation due to underlying diseases. Duff et al. found that the 1-year mortality for individuals with chronic limb ischemia and no amputation was 30%, while those with chronic ischemia and major amputation had a 1-year mortality risk at 41% [1^8^]. Other studies show that patients with a chronic leg wound or diabetic foot ulcer have a reported 5-year mortality risk at 39% and 30.5% respectively [17,19]. These figures suggest that some of the high postoperative mortality could be explained in the extensive comorbidity profile, and the mortality risk may be even higher if patients had not undergone amputation at all.

The results of our study favor initial transtibial amputation in terms of postoperative mortality risk; however, this initial level has a continuously higher re-amputation risk than transfemoral amputation [9]. Therefore, only various considerations including complication risk, mortality risk, and attainable mobility may lead to a balanced and individually tailored decision for each patient.

### Strengths and limitations

Through the Danish health registers, we were able to generate a large study population, which in general results in a minimized risk of selection bias. The Danish health registers contains complete data of high quality, minimizing the risk of information bias.

This study also has some general limitations due to the register-based design. First, the Danish health registers lack information related to lifestyle and detailed information on procedures and results of biochemistry and imaging. With no lifestyle data, e.g., frailty score, physical activity, smoking status, and body mass index, there is a risk of potential confounders that we were not able to adjust for in the analysis. A significant limitation and source of bias is the inability to ascertain the absolute cause of the major amputation, as the DNPR does not contain the indication. Furthermore, infections, sepsis, and acute ischemia, which are the primary indications for considering amputation, invariably lead to increased mortality. Additionally, our analysis does not differentiate between acute and elective patients, presenting another layer of complexity in understanding the outcomes. In addition, the quality of data depends on the providers coding the procedures and diagnoses precisely.

The data from our study cannot state whether initial transtibial amputation is better than transfemoral amputation for the individual patient, but can provide perspective to guide clinicians in deciding on initial amputation level.

### Conclusion

The mortality after major lower extremity amputation declined in the 12-year study period, but the 1-year mortality remained high after both transtibial and transfemoral amputations (20% and 46% in 2021). The mortality in the first year was significantly higher after a transfemoral amputation than after a transtibial amputation at all time points. Additionally, in adjusted analysis, the mortality risk remained higher after transfemoral amputation.

***In perspective,*** major lower extremity amputation patients constitute one of the most fragile orthopedic patient groups, emphasizing an increased need for attention in the pre-, peri-, and postoperative setting. Mortality rates after major amputation remain unacceptably high by modern standards. In response, the Vascular Society of Great Britain and Ireland implemented a quality improvement framework, aiming to reduce 90-day mortality rates to below 10% [20]. This, combined with implementation of orthogeriatric rounds, could serve as a model for reducing and maintaining lower mortality rates after major amputations. Orthogeriatric care for hip fracture patients has demonstrated improved outcomes, including reduced mortality, compared with orthopedic care alone [21-24]. A comprehensive national protocol with an orthogeriatric approach for pre-, peri-, and postoperative care in major lower extremity amputation patients may further decrease mortality rates.

## Supplementary Material



## Supplementary data

Definiations, criteria and Table 3 are available as supplementary data on the article page, doi:10.2340/17453674.2024.40996

## Notes

ATHB, KHR, and MLL designed the study protocol. ATHB conducted the data management and analysis. ATHB drafted the manuscript, first revised critically by MLL, then KHR, TN, and HS afterwards. All authors approved the final manuscript.

The authors acknowledge OPEN Registry & Statistics for their insight in data management and statistics advice.

Handling co-editors: Bart A. Swierstra and Robin Christensen

*Acta* thanks Jaap van Netten for help with peer review of this manuscript.
