# Supplementary material for: Mortality after major lower extremity amputation and association with index level: a cohort study based on 11,205 first-time amputations from nationwide Danish databases
Source: Acta Orthop. 2024 Jun 19;95:358–63. doi: 10.2340/17453674.2024.40996 (PMC11186348; doi:10.2340/17453674.2024.40996)
Supplement: Supplementary file 1 [file ActaO-95-40996-s1.pdf]

Supplementary information

This appendix addresses the definition of variables.

Amputation codes

Primary major amputations

Above knee amputation	
Hip disarticulation	KNFQ09
Transfemoral amputation	KNFQ19
Other amputation on the femur/hip	KNFQ99
Below knee amputation	
Knee disarticulation	KNGQ09
Transtibial amputation	KNGQ19
Other amputation on knee/tibia	KNGQ99
Revision codes – stump revision	
knee/lower leg	KNGQ29
after knee disarticulation	KNGQ29A
after transtibial amputation	KNGQ29B
hip/tight	KNFQ29
after hip disarticulation	KNFQ29A
after transfemoral amputation	KNFQ29B

Prior minor amputation is defined as 1 or more registered procedures before index surgery with procedure code KNHQ* despite TUL codes.

Minor amputations

Foot amputation	KNHQ10–18, KNHQ99, KNHQ00
Toe amputation	KNHQ02–07, KNHQ14

Osseointegration codes are excluded**:** KNGQ39, KNGQ49, KNFQ39, KNFQ49

A case is excluded in case of a revision code without a prior primary amputation code.

KNGQ99/KNFQ99 are grouped as transfemoral amputation (KNFQ19) or transtibial amputation (KNGQ19), respectively.

Sarcoma exclusion criteria: A case is excluded if a sarcoma diagnosis (DC40*, DC41* or DC49*) was registered ±1 year from index surgery.

Trauma exclusion criteria: An amputation is classified as trauma associated, and then excluded if 1 of the following ICD10 codes is present in relation to the amputation: DS980 (traumatic amputation, foot), DS88* (traumatic amputation, knee), DS78* (traumatic amputation, hip), DT136 (traumatic

amputation, lower extremity, unspecified), DT05, DT053-6, DT058-9 (traumatic amputation, in combination), DT036 (sequelae after traumatic amputation, lower extremity).

Prior revascularization procedure

Prior revascularization procedure is defined as 1 or more registered revascularization procedures before index: KPDA*, KPDC*, KPDE*, KPDF*, KPDH*, KPDN*, KPDP*, KPDQ*, KPDT10, KPDT30, KPDU70, KPDU74, KPDU82-84, KPDU87, KPDU99, KPEA*, KPEC*, KPEE*, KPEF*, KPEH*, KPEN*, KPEP*, KPEQ* KPET*, KPEU74, KPEU82-84, KPEU87, KPEU99, KPFE*, KPFH*, KPFN*, KPFP*, KPFT*, KPFQ*, KPFU74, KPFU82-84, KPFU87, KPFU99, KPGH*, KPGU74, KPGU83-84, KPGU87, KPGU99, KPGW99.

Definition for diagnoses, ICD10

Diabetes

A patient is categorized with diabetes if 1 of the following ICD10 codes is registered: E10*, E11*, E13*, E14* or the patient has redeemed 2 or more anti-diabetic medicine prescriptions in the same ATC group with the following ATC codes 5 years before the index date: A10A (insulins and analogs) A10B (blood glucose lowering drugs, excl. insulins), A10X (other drugs used in diabetes).

Hypertension

A patient is categorized with hypertension if 1 of the following ICD10 codes I10*–I15* is registered or the patient has redeemed 2 or more prescriptions in the same ATC group 5 years before the index date: antihypertensives (C02–C03, C07–C09).

Dyslipidemia:

A patient is categorized with dyslipidemia if the ICD10-code E78 is registered, or the patient has redeemed 2 or more prescriptions in the same ATC group 5 years before the index date: C10.

Cardiovascular disease (CVD)

A patient is categorized with CVD if 1 of the following ICD10 codes is registered: I20–I25* (angina, myocardial infarction, and ischemic coronary disease), I50* (incompensatio cordis), I63–I67 (cerebral infarction, occlusion of vertebral and cerebral arteries) or G45–G46 (transient ischemic attack, TCI).

Renal insufficiency

A patient is categorized with renal insufficiency if 1 of the following ICD10 codes is registered: N18*, (chronic kidney disease) or N19* (unspecified kidney failure).

Atherosclerosis/PAD:

Atherosclerosis/peripheral arterial disease (PAD) is defined as 1 or more of the following ICD10 codes: I70, I702, I709, I739A, I739C or I743

Table 3. Absolute proportions of procedures at death within the first year of amputation. Values are count (%)

	Transtibial amputation				Transfemoral amputation			
	No. of proc.	Death at 30 days	90 days	1 year	No. of proc.	Death at 30 days	90 days	1 year
**2010**	381	37 (9.7)	67 (18)	117 (31)	509	138 (27)	203 (40)	279 (55)
**2011**	351	53 (15)	70 (20)	111 (32)	449	113 (25)	162 (36)	221 (49)
**2012**	360	51 (14)	78 (22)	131 (36)	542	125 (23)	201 (37)	272 (50)
**2013**	367	62 (17)	90 (25)	125 (34)	544	136 (25)	174 (32)	247 (45)
**2014**	349	35 (10)	53 (15)	99 (28)	546	123 (23)	188 (34)	269 (49)
**2015**	328	38 (12)	63 (19)	94 (29)	654	136 (21)	211 (32)	305 (47)
**2016**	312	24 (7.7)	47 (15)	75 (24)	696	167 (24)	247 (36)	327 (47)
**2017**	383	46 (12)	74 (19)	126 (33)	657	166 (25)	247 (38)	325 (50)
**2018**	314	35 (11)	48 (15)	80 (26)	672	138 (21)	197 (29)	288 (43)
**2019**	293	23 (7.8)	35 (12)	69 (24)	661	141 (21)	211 (32)	303 (45)
**2020**	263	21 (8.0)	35 (13)	68 (25)	671	142 (21)	213 (32)	315 (47)
**2021**	220	15 (6.8)	23 (11)	45 (21)	683	148 (21)	218 (32)	315 (47)
**Total**	3,921	440 (11)	683 (17)	1,140 (29)	7,284	1,673 (23)	2,472 (34)	3,466 (48)
